# Brainstem Encephalitis in Neuropsychiatric Systemic Lupus Erythematosus Mimicking Malignant Lymphoma: A Case Report and Literature Review

**DOI:** 10.7759/cureus.101551

**Published:** 2026-01-14

**Authors:** Tatsuya Ueno, Shuya Ochiai

**Affiliations:** 1 Neurology, Aomori Prefectural Central Hospital, Aomori, JPN

**Keywords:** brainstem encephalitis, differential diagnosis, magnetic resonance imaging, malignant lymphoma, rhombencephalitis, systemic lupus erythematosus

## Abstract

We report a rare case of neuropsychiatric (NP) systemic lupus erythematosus (NPSLE) manifesting as brainstem encephalitis with generalized lymphadenopathy. A 32-year-old woman developed altered consciousness, fever, and pancytopenia, with diffusion-restricted brainstem lesions and elevated soluble interleukin-2 receptor (sIL-2R) levels in both serum and cerebrospinal fluid (CSF), accompanied by systemic lymphadenopathy and cytopenia, strongly mimicking malignant lymphoma. Histopathology excluded malignancy. The patient underwent immunosuppressive therapy with corticosteroids and cyclophosphamide, leading to marked neurological improvement. This case highlights the diagnostic challenge in differentiating NPSLE with brainstem involvement from lymphoproliferative disorders and underscores the importance of early biopsy and prompt immunosuppressive intervention to achieve favorable outcomes.

## Introduction

Systemic lupus erythematosus (SLE) demonstrates substantial global epidemiologic variability, with incidence rates ranging between 1.5 and 11.0 per 100,000 person-years and prevalence of 13.0-7713.5 per 100,000 individuals [1]. Neuropsychiatric (NP) manifestations occur in approximately 56.3% of patients with SLE (95% confidence interval, 42.5-74.7%), as reported in a meta-analysis incorporating all American College of Rheumatology-defined NP syndromes, and typically emerge within the first year after diagnosis [2,3]. NPSLE is considered a heterogeneous syndrome involving multiple pathogenic mechanisms, including immune complex-mediated vasculopathy, autoantibody-induced neuronal dysfunction, and cytokine-driven neuroinflammation [2-4]. These mechanisms result in a broad clinical spectrum ranging between mild cognitive or mood disturbances and severe inflammatory encephalitis and focal neurological deficits [2-4]. NPSLE contributes to significant morbidity and mortality [2,4]. Common manifestations include headache, mood disorders, and seizures [2]; however, brainstem involvement remains exceptionally rare [5-10]. Reported cases of NPSLE with brainstem lesions have shown relatively consistent clinical patterns, including altered consciousness, cranial nerve-related symptoms, motor weakness, and systemic lupus activity such as fever, cytopenia, and hypocomplementemia [5-10].

Brainstem encephalitis has a broad differential diagnosis, particularly in patients with autoimmune disease, including autoimmune encephalitis (e.g., Bickerstaff brainstem encephalitis), neurosarcoidosis, demyelinating disorders, infectious rhombencephalitis, and central nervous system (CNS) lymphoma. In this clinical context, distinguishing NPSLE from lymphoproliferative disorders poses a considerable diagnostic challenge, particularly when systemic abnormalities demonstrate significant overlap. The constellation of generalized lymphadenopathy, pancytopenia, and elevated soluble interleukin-2 receptor (sIL-2R) levels, findings characteristically associated with malignant lymphoma, may closely mimic lymphoproliferative disease. Furthermore, the presence of diffusion-restricted lesions within the brainstem on magnetic resonance imaging (MRI) may heighten clinical suspicion of neoplastic infiltration, thereby necessitating expeditious histopathological confirmation to prevent delays in appropriate therapeutic intervention.

Herein, we report the case of a 32-year-old woman with NPSLE manifesting as brainstem encephalitis who exhibited altered consciousness, fever, pancytopenia, and diffuse lymphadenopathy with neuroimaging abnormalities in the brainstem. A rapid differentiation from malignant lymphoma was made; the patient favorably responded to treatment. We also provide a literature review of brainstem encephalitis in patients with NPSLE.

## Case presentation

A 32-year-old woman with a five-year history of Sjögren syndrome on prednisolone (10 mg/day) presented with a one-day history of fever and altered consciousness. She developed joint stiffness two months prior, followed by low-grade fever and malaise one month before admission. On admission, her vital signs were: temperature, 38.0°C; blood pressure, 126/83 mmHg; heart rate, 108 beats/min; and oxygen saturation, 100% on room air. Physical examination revealed lymphadenopathy in the cervical, bilateral axillary, and inguinal regions, with nodes measuring 1-2 cm in size, elastic in consistency, and mobile. Neurological examination revealed a Glasgow Coma Scale score of 11 (E3V3M5). Cranial nerve examination showed equal, round, and reactive pupils with brisk light reflexes; extraocular movements were preserved, without ptosis, nystagmus, or diplopia. Furthermore, although evaluation of visual fields, hearing, swallowing function, and facial sensation was limited due to impaired consciousness, speech articulation was intact without dysarthria. Motor examination demonstrated bilateral limb weakness with spasticity (Medical Research Council grade 4/5 distally and 3/5 proximally), precluding reliable cerebellar testing. Deep tendon reflexes were brisk bilaterally with bilateral Babinski signs; sensory examination was unreliable. Moreover, the patient was unable to ambulate at presentation, corresponding to an initial modified Rankin Scale score of 5. No meningeal signs were present. Laboratory studies showed pancytopenia (white blood cell, 2.7 × 10³/μL; hemoglobin, 9.5 g/dL; platelets, 12.5 × 10⁴/μL), elevated lactate dehydrogenase level (1818 U/L; reference range, 124-222 U/L), elevated inflammatory marker levels (CRP, 9.35 mg/dL; reference range, 0.0-0.14 mg/dL), elevated ferritin (5629.6 ng/mL; reference range, 4.1-120.2 ng/mL) and sIL-2R levels (2689 U/mL; reference range, 121-613 U/mL), and decreased complement consumption (C3, 26.8 mg/dL; reference range, 73.0-138.0 mg/dL; C4, 12.7 mg/dL; reference range: 11.0-31.0 mg/dL; and CH50, 14.9 U/mL; reference range: 31.6-57.6 U/mL). Antinuclear antibody (1:1280; speckled pattern), anti-SS-A antibody (>1200 U/mL), and anti-Sm antibody (>600 U/mL) tests were positive. Tests for anti-dsDNA and antiphospholipid antibodies were negative. Urinalysis revealed no abnormalities. Cerebrospinal fluid (CSF) analysis showed pleocytosis (12 cells/mm³, predominantly mononuclear), elevated protein level (139 mg/dL), elevated sIL-2R level (174 U/mL), elevated IL-6 level (257 pg/mL), and low glucose level (29 mg/dL), with normal blood glucose level (95 mg/dL). The IgG index was 0.91, and oligoclonal band tests were negative. Meningitis/encephalitis panel and cultures were negative (Table 1).

**Table 1:** Laboratory parameters CH50, total hemolytic complement activity; sIL-2R, soluble interleukin-2 receptor; IL-6, interleukin-6; CSF, cerebral spinal fluid; IgG, immunoglobulin G

Parameters	Patient values	Reference range
Serum		
White blood cells (/μL)	2.7 × 10³	3.3-8.6 × 10³
Hemoglobin (g/dL)	9.5	11.6-14.8
Platelets (/μL)	12.5 × 10⁴	15.8-34.8 × 10⁴
Lactate dehydrogenase (U/L)	1818	124-222
C-reactive protein (mg/dL)	9.35	0.0-0.14
Ferritin (ng/mL)	5629.6	4.1-120.2
sIL-2R (U/mL)	2689	121-613
C3 (mg/dL)	26.8	73.0-138.0
C4 (mg/dL)	12.7	11.0-31.0
CH50 (U/mL)	14.9	31.6-57.6
Antinuclear antibody	1:1280, speckled pattern	-
Anti-SS-A antibody (U/mL)	>1200	<10
Anti-Sm antibody (U/mL)	>600	<10
Anti-dsDNA antibody (IU/mL)	4.9	<12
Antiphospholipid antibody	Negative	-
Anti-U1 ribonucleoprotein antibody (IU/mL)	>550	<10
Anti-ribosomal P protein antibody	Negative	-
Glucose (mg/dL)	95	73-109
CSF		
Color	-	-
CSF pressure (mmH2O)	-	70-180
White blood cells (cells/mm³)	12	<5
Polymorphonuclear leukocyte	1	-
Mononuclear leukocyte	11	-
Protein (mg/dL)	139	15-45
Glucose (mg/dL)	29	40-70
sIL-2R (U/mL)	174	<50
IL-6 (pg/mL)	257	<4.3
IgG index	0.91	<0.7
Oligoclonal band	Negative	-
Anti-Sm antibody (U/mL)	19.6	-
Anti-U1 ribonucleoprotein antibody (IU/mL)	55.7	-
Anti-ribosomal P protein antibody	Negative	-
Anti-N-methyl-D-aspartate receptor antibody	Negative	-

**Figure 1:**
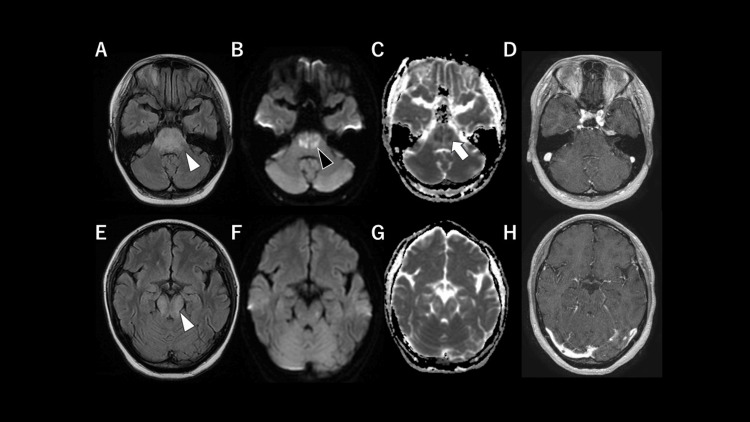
Neuroimaging at presentation (A,E) Fluid-attenuated inversion recovery sequences showing symmetric hyperintense lesions with swelling from the pons to the midbrain (white arrowhead). (B,F) Diffusion-weighted imaging revealing scattered hyperintense lesions with corresponding distribution (black arrowhead), showing restricted diffusion on apparent diffusion coefficient maps (C,G) (white arrow). (D,H) Post-gadolinium T1-weighted fast spoiled gradient-recalled echo images showing no abnormal contrast enhancement.

Additional antibody testing showed positivity for anti-Sm and anti-U1 ribonucleoprotein in serum and CSF, while showing negativity for anti-ribosomal P protein and anti-N-methyl-D-aspartate receptor antibodies (Table 1). MRI showed symmetric fluid-attenuated inversion recovery hyperintensity and swelling from the pons to the midbrain, with diffusion-weighted imaging (DWI) hyperintensity and apparent diffusion coefficient (ADC) hypointensity, without contrast enhancement (Figure 1).

**Figure 2:**
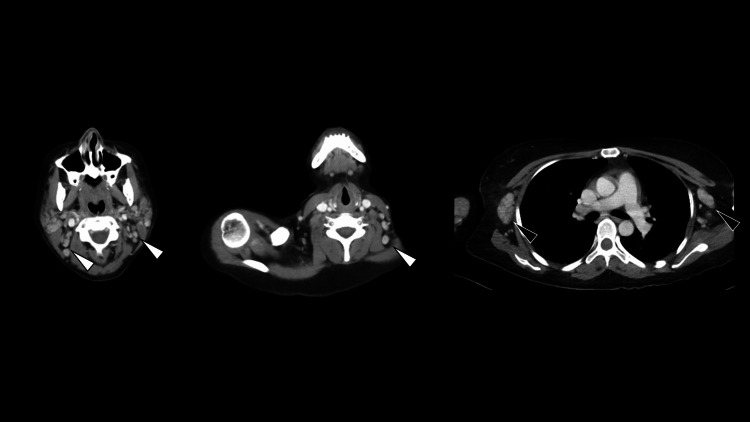
Contrast-enhanced computed tomography of the whole body at presentation Computed tomography demonstrates generalized lymphadenopathy in cervical (white arrowheads) and axillary regions (black arrowheads).

Computed tomography revealed generalized lymphadenopathy (Figure 2).

The provisional diagnosis on admission was suspected malignant lymphoma; however, infectious brainstem encephalitis and autoimmune brainstem encephalitis were also considered as differential diagnoses. Empirical treatment with meropenem (6 g/day) and acyclovir (1500 mg/day) was initiated on day 1, and intravenous methylprednisolone pulse therapy (1 g/day for three consecutive days) was added on day two. Bone marrow aspiration and cervical lymph node biopsy on days two and four showed reactive changes without evidence of malignancy, excluding malignant lymphoma. The patient’s altered consciousness persisted without improvement through day seven. On day eight, she developed a discoid rash and an oral ulcer. Based on the absence of histopathological evidence for lymphoma and the 2019 European League Against Rheumatism/American College of Rheumatology criteria [11], SLE with CNS involvement was diagnosed as NPSLE. Specifically, the patient met the entry criterion of antinuclear antibody positivity with an accumulated total score of 20 points, including fever (3 points), leukopenia (3 points), thrombocytopenia (4 points), low complement C3 levels (3 points), oral ulcers (2 points), and anti-Sm antibody positivity (6 points), thereby exceeding the classification threshold of ≥10 points. Cyclophosphamide (500 mg/day) initiated on day 10, followed by oral prednisolone (50 mg daily) from day 20, led to rapid clinical improvement. Consciousness normalized within days, and a follow-up MRI on day 21 showed a marked reduction in brainstem swelling. Hydroxychloroquine (200 mg/day) treatment began on day 36. Although mild lower extremity muscle weakness persisted, the patient’s ambulatory status stabilized, and she was discharged on day 68. At follow-up on day 99, her gait continued to improve, and her functional outcome reached a modified Rankin Scale score of 2.

## Discussion

This case highlights the diagnostic challenge in distinguishing NPSLE with brainstem involvement from malignancy. Fever, pancytopenia, diffuse lymphadenopathy, elevated serum and CSF sIL-2R levels, and diffusion-restricted brainstem lesions initially raised suspicion of malignant lymphoma; however, histopathological evaluation revealed only reactive changes. In addition, infectious and autoimmune encephalitis were excluded based on CSF analysis results. These findings, together with inflammatory CSF markers and non-enhancing MRI lesions, supported immune-mediated neuroinflammation and justified prompt immunosuppressive therapy. To the best of our knowledge, this is the first reported case of NPSLE manifesting as brainstem encephalitis with generalized lymphadenopathy and concurrent elevation of sIL-2R level in both serum and CSF. Extended neuronal antibody testing was not performed, as the clinical presentation, imaging findings, and disease course were not suggestive of other autoimmune or paraneoplastic encephalitis.

It is important to note that sIL-2R is a marker of lymphocyte activation and is not specific to lymphoma; elevated levels have also been reported to correlate with disease activity in SLE [12,13]. Accordingly, the marked elevation of serum and CSF sIL-2R in our patient contributed to diagnostic uncertainty but did not by itself establish a lymphoproliferative disorder. Although CSF biomarkers such as sIL-2R and cytokines can be informative in CNS lymphoma, these markers may overlap with severe inflammatory conditions [14], and lymphoma was ultimately excluded by histopathology and lack of contrast enhancement. In addition, hypoglycorrhachia raised concern for infection or malignancy; however, low CSF glucose has been described in SLE-associated chronic meningitis [15], and in our case, microbiological studies were negative and neurological deficits improved with immunosuppressive therapy, supporting immune-mediated neuroinflammation.

Including this case, only seven cases of NPSLE with brainstem lesions have been reported, all in women aged 19-33 years (Table 2) [5-10].

**Table 2:** Clinical features of patients with NPSLE involving the brainstem AFB, acid-fast bacilli; ANA, antinuclear antibody; CE, contrast enhancement; CSF, cerebrospinal fluid; CRP, C-reactive protein; DTR, deep tendon reflex; ESR, erythrocyte sedimentation rate; DWI, diffusion-weighted imaging; GCS, Glasgow Coma Scale; IVCY, intravenous cyclophosphamide; IVIg, intravenous immunoglobulins; IVMP, intravenous methylprednisolone; NPSLE, neuropsychiatric systemic lupus erythematosus

Reference	Age/sex	Pre-NPSLE symptoms	Symptoms of NPSLE	Time from prodromal symptoms to NPSLE onset	Fever	Physical examination	Neurological examination	Labo-data	CSF	MRI lesion	DWI	CE	Treatment	Prognosis
Kumar et al., 2009 [5]	19/F	Fever, loss of appetite, loss of weight, erythematous rash, and photosensitivity	Altered mental status	2 months	NA	Erythematous plaques	GCS of 10 (E4M5V1) and right hemiparesis	Decreased hemoglobin level (7.9 g/dL), decreased complement levels (C3/C4), and positive ANA in speckled pattern (3+)	No cells and elevated protein level (249 mg/dL)	Posterior limbs of bilateral internal capsules, midbrain, pons, medulla oblongata, bilateral middle cerebellar peduncles, dentate nuclei, and bilateral hypothalami	No restriction	NA	IVMP and IVCY	Completely improved
Hu et al., 2017 [6]	19/F	Recurring joint swelling, chilblains, and alopecia	Fever, headache, weakness, sleepiness, dysarthria, and dysphagia	7 days	37.8	Normal	Neck stiffness, horizontal nystagmus, limited vertical eye movement, lower extremity-predominant weakness, hypotonia, hyperesthesia, and absent DTR in the lower limbs	Increased ESR (72 mm/h), elevated CRP (44.50 mg/L), elevated ANA titer (1:640 with speckled pattern), elevated anti-dsDNA antibody level (676.21 IU/mL), and decreased complement C3 level (0.47 g/L)	Increased leukocyte count (110 × 10⁶/L) and increased protein level (775.79 mg/L)	Medulla oblongata and spinal cord	NA	NA	IVMP, IVIg, and IVCY	The neurological deficit was completely resolved
Polhemus et al., 2024 [7]	33/F	NA	Fever, generalized weakness, headache, nausea, and vomiting	No prodromal phase	40.6	NA	Unable to follow commands, vertical downbeat nystagmus, limited upward gaze with horizontal gaze palsy, and hyperreflexia quadriparesis	Elevated ANA titer (1:2560) and ANA HEp-2 IgG antibodies positive	Pleocytosis (320 nucleated cells/μL), elevated protein (244 mg/dL), and normal glucose (63 mg/dL)	Pons	High signal intensity in some areas	Partial	IVMP, IVIg, IVCY, and hydroxychloroquine	Partially improved
Khan et al., 2024 [8]	20/F	Fever	Seizure and erythematous papular eruptions	18 days	NA	Hyperpigmented crusted lesions, facial puffiness, and periorbital and eyelid swelling	GCS of 7/15	Positive ANA and low complement levels (C3 and C4)	Negative for AFB, fungi, and other pathogens	Temporal lobes, left thalamus, right hippocampus, parahippocampal region, left cerebellar hemisphere, and the pons	NA	NA	Steroid and cyclophosphamide	Partially improved
Branch et al., 2024 [9]	22/F	None	Lethargy, bilateral arm shaking, and altered mental status	2 weeks	NA	NA	Decreased level of consciousness and upper extremity spasticity	Pancytopenia positive ANA (1:5120), positive anti-dsDNA, and low C3/C4	NA	Brainstem, thalamus, and striatum	Punctate striatal diffusion restriction	NA	Corticosteroids, cyclophosphamide, and hydroxychloroquine	Only improvements to the image are listed
Shiratani et al., 2025 [10]	18/F	Alopecia, facial rashes, fever, and polyarthralgia	Thunderclap headache, hypothermia, hoarseness, and dysphagia	8 months	38.2	Malar rash and alopecia areata	Left vocal cord paralysis, nystagmus in the left gaze, right curtain sign, thermal hypoalgesia of the right upper limb, lagophthalmos, and left upper and lower facial weakness	Leukopenia (2800/μL), thrombocytopenia (114 × 10^3^/μL), positive ANA (1:80), elevated anti-dsDNA antibody level (44.2 IU/mL), decreased complement C3 level (32.8 mg/dL), and decreased complement C4 level (3.6 mg/dL)	Normal cell, elevated protein level (67.8 mg/dL), and elevated IL-6 level (910 pg/mL)	Dorsal pons and medulla oblongata	NA	Normal	IVMP, IVCY, belimumab, and plasmapheresis	Hoarseness gradually improved and dysphagia gradually improved
Present case	32/F	Joint stiffness, fever, and malaise	Altered consciousness	2 months	38	Lymphadenopathy	GCS of 11 (E3V3M5), bilateral limb weakness with spasticity, brisk DTR, and bilateral Babinski signs	Pancytopenia, elevated CRP (9.35 mg/dL), elevated ferritin (5629.6 ng/mL), elevated sIL-2R (2689 U/mL), decreased complement C3 level (26.8 mg/dL), elevated ANA titer (1:1280 with speckled pattern), and elevated anti-Sm antibody (>600 U/mL)	Pleocytosis (12 cells/mm³), elevated protein (139 mg/dL), elevated sIL-2R (174 U/mL), elevated IL-6 (257 pg/mL), low glucose (29 mg/dL), elevated IgG index (0.91), and negative oligoclonal bands	Midbrain and pons	Scattered hyperintense lesions	No	IVMP and IVCY	Partially improved

The time interval from prodromal symptoms to NPSLE onset varied considerably, ranging from cases wherein NP manifestations were the initial presenting feature to cases of systemic SLE symptoms appearing up to eight months prior. The most frequent systemic symptoms before the onset of NP manifestations were fever (57%), alopecia (29%), joint symptoms (29%), and facial rashes or photosensitivity (29%). The predominant neurological manifestations were altered consciousness (57%), limb weakness (43%), and ocular movement abnormalities or nystagmus (29%). Joint stiffness, fever, and malaise preceded altered consciousness and limb weakness (Table 2). The presence of prominent lymphadenopathy in our case represented a distinctive feature not documented in previous reports. The most consistent laboratory abnormalities were positive antinuclear antibody (ANA, 100%), low complement levels (86%), and positive anti-dsDNA antibodies (71%), contributing to the diagnosis of SLE (Table 2). The most consistent findings were elevated CSF protein levels (86%) and pleocytosis (57%). Laboratory investigations revealed antinuclear antibody positivity, reduced complement levels, CSF pleocytosis, and increased CSF protein levels. Tests were negative for anti-dsDNA antibodies but positive for anti-Sm antibodies. Pancytopenia and elevated sIL-2R levels in both serum and CSF represented distinctive features. Simultaneous elevation in both serum and CSF sIL-2R levels has not been reported in patients with NPSLE presenting with brainstem encephalitis (Table 2) [5-10]. Bone marrow aspiration and lymph node biopsy revealed reactive changes without neoplastic infiltration, confirming that lymphadenopathy and inflammatory changes were secondary to lupus-related immune activation. Brainstem MRI abnormalities in NPSLE cases most frequently involved the pons (57%), followed by the midbrain (43%), and medulla oblongata (43%) (Table 2) [5-10]. Abnormal DWI findings appeared as patchy or punctate hyperintense lesions in the pons, midbrain, or striatum [7,9]. Only one case (14%) demonstrated partial enhancement within the pontine lesion [7]. In the present case, fluid-attenuated inversion recovery hyperintensity extended from the midbrain to the pons without contrast enhancement; moreover, DWI hyperintensity was noted, consistent with previous reports. DWI hyperintensity with reduced ADC values generally reflects restricted diffusion due to cytotoxic edema or high cellularity, as commonly observed in cases of lymphoma or severe inflammation [16]. In primary CNS lymphoma, densely packed tumor cells restrict water diffusion, leading to hyperintensity on DWI and low ADC values; however, when lymphoma cells are sparsely distributed within the perivascular spaces, diffusion restriction may be less apparent [17]. In this case, DWI hyperintensity with reduced ADC values reflected restricted diffusion due to cytotoxic edema and increased cellularity, suggesting a more severe pathological condition, including lymphoma, infection, severe inflammation, and ischemia [16]. Meanwhile, the absence of contrast enhancement and favorable response to immunosuppressive therapy indicated that severe inflammatory activity was likely involved rather than neoplastic infiltration.

All patients received high-dose intravenous methylprednisolone as first-line therapy. Most patients (86%) were treated with cyclophosphamide, the primary immunosuppressive agent for CNS lupus (Table 2) [5-10]. Most patients achieved favorable outcomes, from partial to complete neurological recovery. In patients with incomplete recovery, DWI frequently demonstrated restricted diffusion, which might indicate more severe brainstem involvement and reflect a poorer neurological prognosis [7,9]. Our patient received intravenous methylprednisolone pulse therapy and cyclophosphamide, which resulted in incomplete recovery characterized by mild muscle weakness in both lower extremities.

This study has some limitations. The patient’s complete medical history prior to Sjögren syndrome diagnosis was not fully accessible, which might have influenced the interpretation of disease progression. The relatively short follow-up period (99 days) also limited our ability to assess long-term neurological outcomes and potential disease relapse. Additionally, as a single case report, the generalizability of the findings is limited.

## Conclusions

This case highlights the diagnostic complexity of NPSLE presenting with brainstem encephalitis and generalized lymphadenopathy. Simultaneous elevation of sIL-2R in both serum and CSF, together with diffusion-restricted brainstem lesions on MRI, mimicked malignant lymphoma. This underscores the importance of early histopathological confirmation to avoid treatment delay. To the best of our knowledge, no previous cases of NPSLE with brainstem encephalitis showing simultaneous serum and CSF sIL-2R elevation have been reported. Furthermore, the generalized lymphadenopathy in this case was attributed to lupus-related immune activation rather than neoplastic infiltration.
